# A chromosome-level reference genome of the wax gourd (*Benincasa hispida*)

**DOI:** 10.1038/s41597-023-01986-7

**Published:** 2023-02-07

**Authors:** Wenlong Luo, Jinqiang Yan, Shanwei Luo, Wenrui Liu, Dasen Xie, Biao Jiang

**Affiliations:** Guangdong Key Laboratory for New Technology Research of Vegetables, Vegetable Research Institute, Guangdong Academy of Agricultural Sciences, Guangzhou, 510640 China

**Keywords:** Genomics, Plant genetics, Agriculture, Sequencing

## Abstract

The wax gourd (*Benincasa hispida*), the only species in the genus *Benincasa*, is an important crop native to Asia that has been widely planted for multi-purpose uses. The first wax gourd draft genome was published three years ago, but it was incomplete and highly-fragmented due to data and technical limitations. Herein, we report a new chromosome-level genome assembly and annotation of *B. hispida*. We generated 974.87 Mb of unitigs with N50 size of 2.43 Mb via a hybrid assembly strategy by using PacBio long reads and Illumina short reads. We then joined them into scaffolds with Hi-C data, resulting 1862 scaffolds with a total length of 975.62 Mb, and 94.92% of the length (926.05 Mb) is contained in the 12 largest scaffolds corresponding to the 12 chromosomes of *B. hispida*. We predicted 37,092 protein-coding genes, and 85.05% of them were functionally annotated. This chromosome-level reference genome provides significant improvement to the earlier version of draft genome and would be valuable resource for research and molecular breeding of the wax gourd.

## Background & Summary

The wax gourd (*Benincasa hispida*), also known as ash gourd, white gourd, Chinese watermelon or winter gourd, is the only species in the genus *Benincasa*. It is an annual cucurbits native to Asia, and has been used as a vegetable and herbal medicine for thousands of years in China and India^1,2^. In the recent decades, the wax gourd is grown in more and more areas throughout the world for multi-purpose uses. It contains many important nutrients, and some metabolites can be used in treating fever and various disorders^3,4^. Commonly, it is used as an important vegetable and its young leaves, flower buds, immature and mature fruit is cooked and eaten. For its medicinal properties, it has been recognized in the traditional Chinese medicine and Ayurvedic medicine system over thousands of years, and now there are an increasing number of studies reported its medicinal values^5,6^. Moreover, the wax gourd is widely used in the food industry for making candied fruit, moon cakes and many kinds of pies as a base filling material. It is of great importance to broaden our knowledge of the wax gourd for promoting to fully exploit its benefits.

Development of a high-quality reference genome would be very useful for molecular genetics, molecular breeding and evolutionary studies of the wax gourd. Previously, we have reported a draft genome sequence of the wax gourd variety B227, and we revealed that the 12 chromosomes of wax gourd represent the most ancestral karyotype of the investigated cucurbits^1^. The B227 assembly is the only published *de novo* assembly of the wax gourd genome so far. Its contigs and scaffolds were constructed based on Illumina paired-end reads (approx. 28-fold), mate-pair reads (approx. 12-fold) and PacBio long-reads (approx. 15-fold), and the final pseudo-chromosomes were generated by anchoring scaffolds onto a published genetic map^7^. Though developed by combing data from multi platforms, it remains incomplete and highly-fragmented (contig N50 of 68.5 Kb, scaffold N50 of 3.4 Mb), and may contain mis-ordered or mis-oriented scaffolds due to technical limitations of the genetic map based pseudo-chromosome construction as reported in some plant species^8–10^. There is still much left to be improved about the wax gourd reference genome, and the availability of genomes of different varieties would provide more resources that can help to understand the genetic variations and evolutionary history of the crop.

**Fig. 1:**
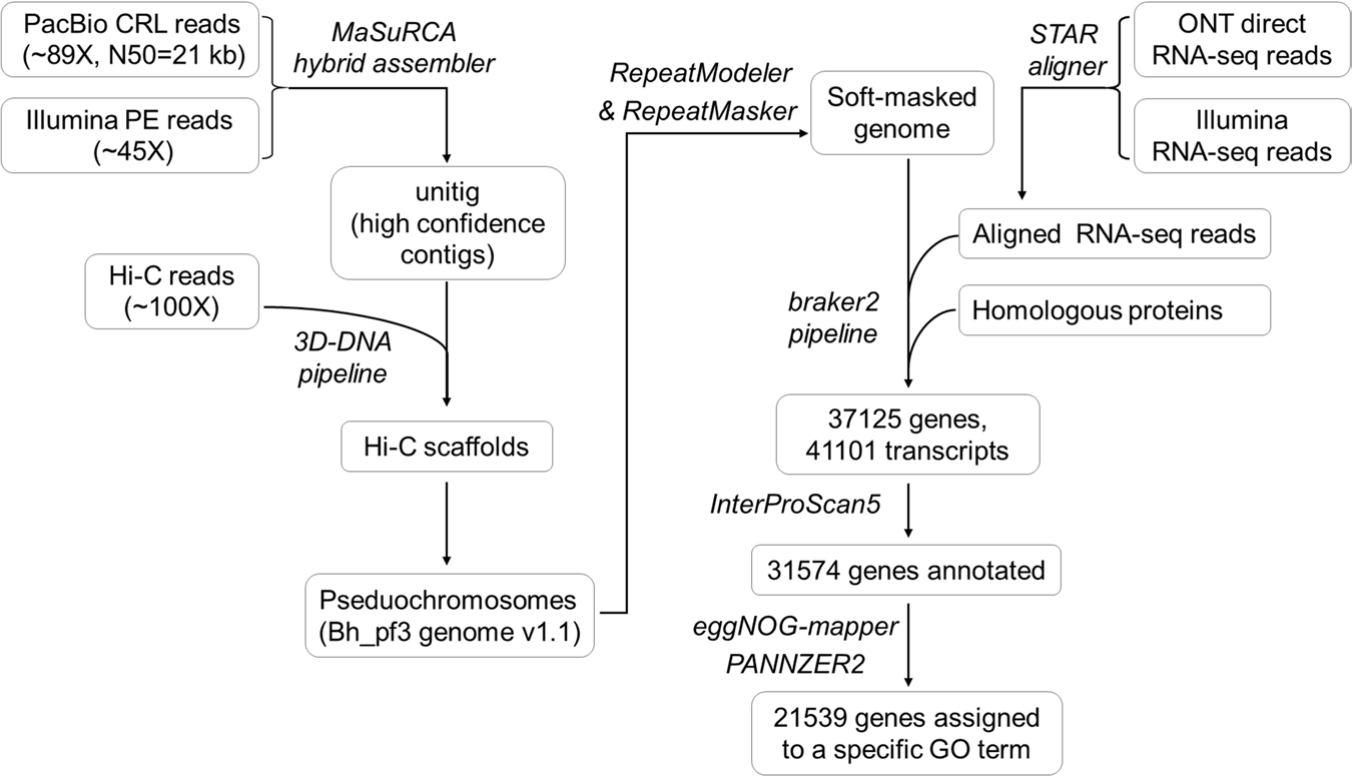
Overview of genome sequencing, assembly and annotation. Data information is shown in rectangles, software and tools are indicated in italic.

Through our continuous efforts, a high-quality and near-complete reference genome assembly has been achieved. Herein, we report a chromosome-scale high quality genome assembly of the wax gourd variety pf3 by combined use of high-coverage PacBio long reads (approx. 86-fold), Illumina short reads (approx. 50-fold) and the Hi-C data. The *de novo* genome assembly and annotation workflow is as showed in the Fig. 1.

## Methods

### Sample selection, library preparation and sequencing

**Fig. 2:**
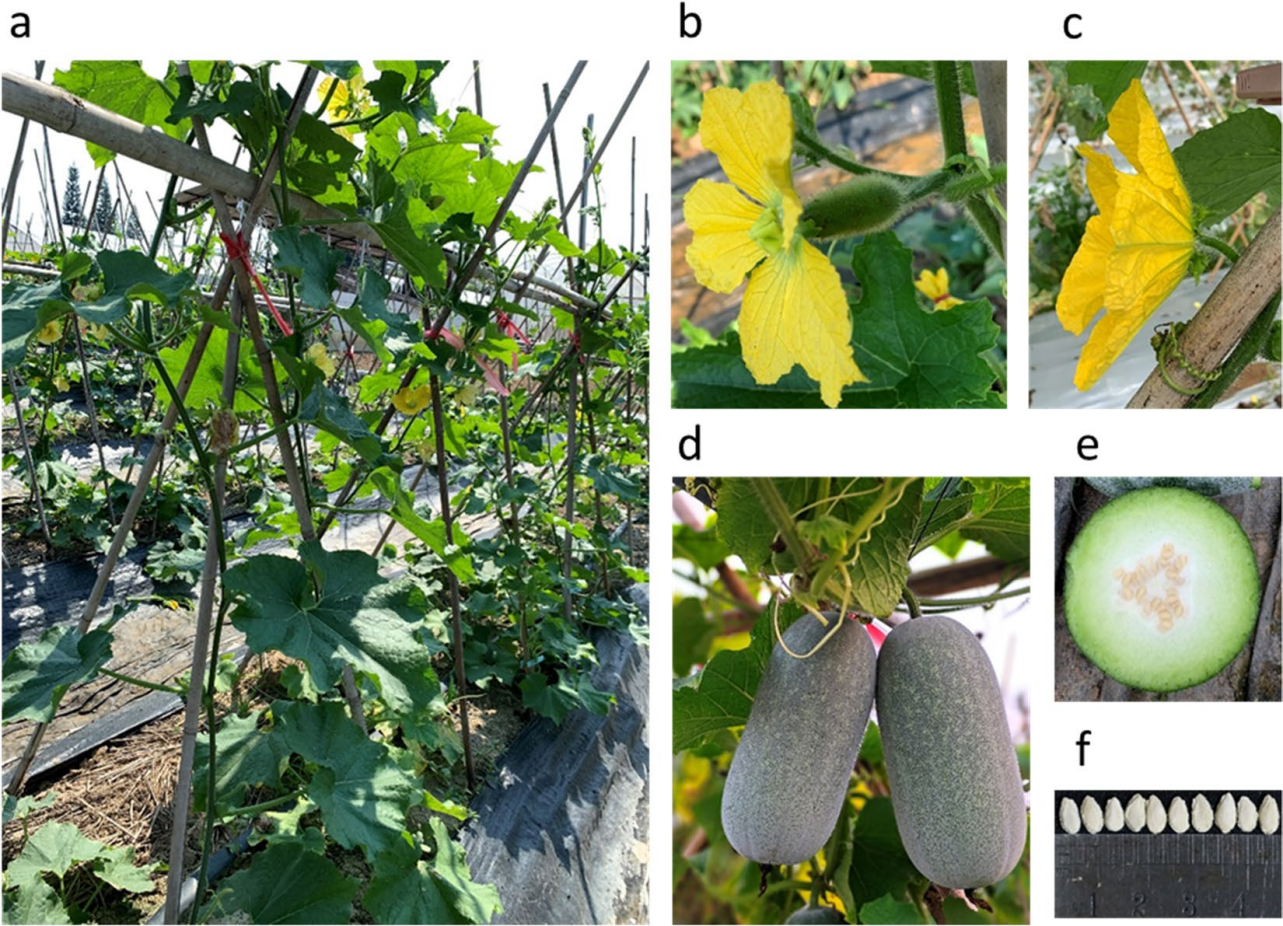
Morphology of the sequenced wax gourd cv. pf3. (**a**) The whole plant in the field. (**b**) Female flower. (**c**) Male flower. (**d**) Mature fruit. (**e**) Transection of mature fruit. (**f**) Seeds.

Previously we divided wax gourd germplasm into four groups according to their genomic variation data^1^, which were the wild group (W), the landrace (L), the two cultivated groups (C1 and C2). The wax gourd variety pf3 used for sequencing in this study is an inbreed line developed by us at the Vegetable Research Institute, Guangdong Academy of Agricultural Sciences in Guangzhou, China. It derived from a cross between a small fruit landrace (belongs to L group) collected from Yunnan and a giant fruit elite line (belongs to C2 group). It shows moderate fruit size with wax, high-yielding potential and good quality of taste (detailed morphology see Fig. 2). Fresh seedlings of the pf3 were used for high-quality DNA extraction followed by construction of PacBio SMRT Bell library, Illumina short-read library and Hi-C library. The Bell library was sequenced on the PacBio Sequel II platform (CLR mode), and then the output raw subreads bam file was converted to fastq format, generating 86.53 Gb data (Table 1). The Illumina short-read and Hi-C library was sequenced on the Illumina NovaSeq-6000 platform(PE150), generating 50.92 Gb and 99.55 Gb clean data respectively. All the DNA extraction, library construction and sequencing procedures were performed by the Novogene Company (Tianjin, China) according to the manufacturer’s protocols.

**Table 1:** Statistics of the sequencing data of the wax gourd variaty pf3.

Library types	Sample	Molecule	Platform	Insert size	Data size (Gb)	Application
SMRT Bell	Seedlings	DNA	PacBio Sequel II	>20 kb	86.53	Unitig assembly
Short-read	Seedlings	DNA	Illumina NovaSeq 6000	300–500 bp	50.92	Unitig assembly
Hi-C	Seedlings	DNA	Illumina NovaSeq 6000	300–500 bp	99.55	Scaffolding and pseudo-chromosome construction
Direct-cDNA	Root, stem, leaf and flower	RNA	Nanopore PromethION	RNA length	50.05	Structural annotation
TruSeq-cDNA	Root, stem, leaf and flower	RNA	Illumina Hiseq 4000	RNA length	8.33	Structural annotation

### RNA sequencing

Root, stem, leaf and flower tissue of the pf3 plants were collected for RNA extraction. Total RNA was extracted from each tissue respectively, and then equal amount of them were pooled together. Thereafter, direct-cDNA sequencing and TruSeq RNA-seq library were constructed using the pooled RNA, and the transcriptomes were sequenced on the Nanopore PromethION and Illumina Hiseq4000 platform by Novogene Company (Tianjin, China), respectively. In total, 50.05 Gb full-length RNA-seq data and 8.33 Gb short-read RNA-seq data were obtained (Table 1). These RNA-seq data were used for whole-genome protein-coding gene prediction.

### *De novo* genome assembly

**Fig. 3:**
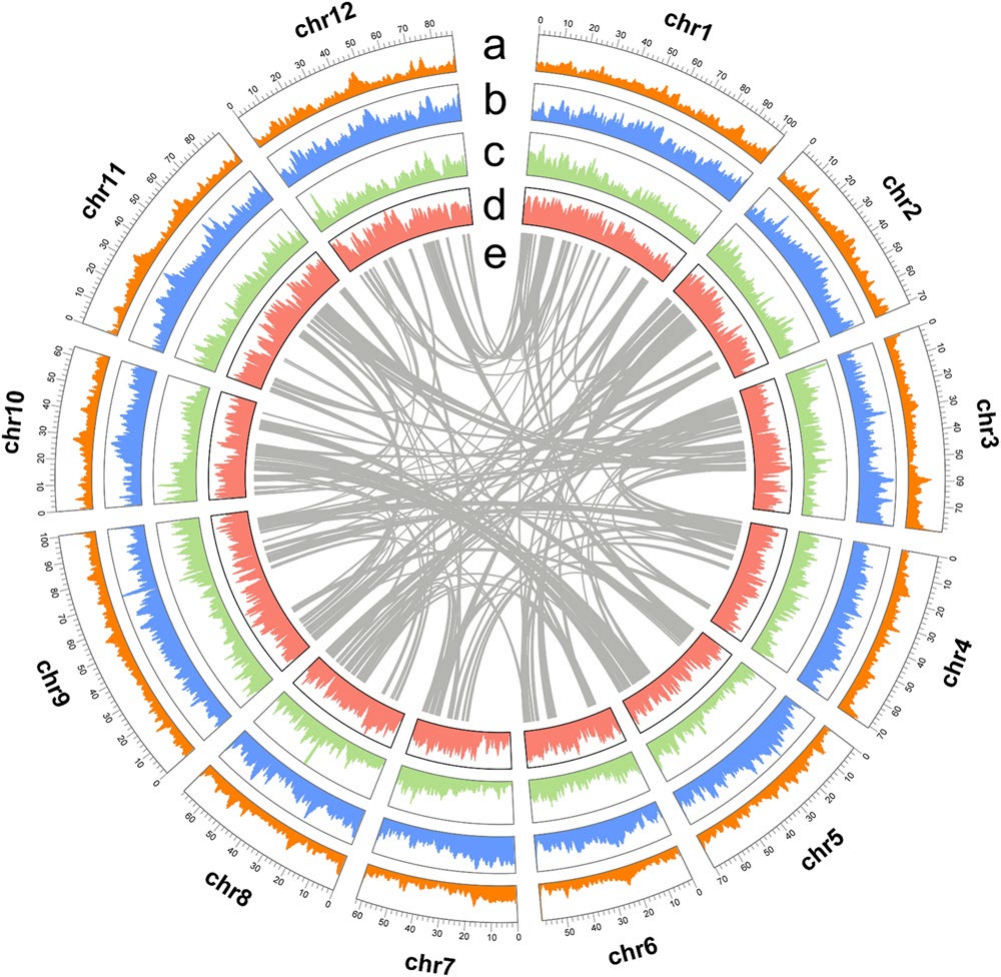
Genome features of the wax gourd cv. pf3. (**a**) GC content (30–50%) across 12 chromosomes. (**b**) Repeat percentage (60–100%). (**c**) Gene density (0–1452). (**d**) SNP density (0–24764). (**e**) Syntenic blocks of paralogous genes. a-d are drawn in non-overlapping 1 Mb sliding window.

We first converted raw subreads bam file generated by PacBio sequencer into fastq format using the software BAM2fastx. Statistical analysis showed that the average length of the long reads was 17.68 kb, and the N50 length was 21.01 kb. Then we constructed a primary assembly by using MaSuRCA assembler v4.0.9^11^ with default parameters. The assembler built unitigs (high-confidence contigs) through hybrid assembly strategy with high-coverage PacBio long-read and Illumina short-read data, and generated 1897 unitigs with a total size of 974.87 Mb and unitig N50 of 2.43 Mb (Table 2). We then joined unitigs into scaffolds using Hi-C data via Juicer v1.6 and 3D-DNA v180922 pipeline^12^ with default parameters. We further visualized the raw scaffolds and conducted manual curation using the Juicebox tool package v1.22.01^13^. After curation, we obtained 1862 high-accuracy scaffolds with a total length of 975.62 Mb and N50 scaffold size of 70.97 Mb. We designated the assembly pf3 v1.1, and 94.92% of the total length (926.05 Mb) is contained in the 12 largest scaffolds that corresponding to the expected 12 chromosomes of the wax gourd (Fig. 3). By comparison with the B227 assembly we reported previously, great improvement on continuity (contig N50 size of 2.43 Mb vs 68.5 Kb), completeness (975.62 Mb vs 912.95 Mb) and chromosome-anchored size (926.05 Mb vs 859.0 Mb) was achieved in the pf3 v1.1 assembly (Table 2).

**Table 2:** Summary of comparisons of pf3 v1.1 assembly and B227 assembly.

Type	pf3 v1.1	B227
Total size of assembled scaffolds	975.62 Mb	912.95 Mb
GC content	34.94%	34.85%
Number of scaffolds	1,862	2,197
Largest scaffold	102.24 Mb	14.5 Mb
Scaffold N50	70.97 Mb	3.4 Mb
Scaffold L50	6	Not available
N’s per 100 kbp	77.03	1,618.82
Total size of assembled contigs	974.87 Mb	898.17 Mb
Number of contigs	1,897	26,315
Largest contig	24.91 Mb	Not available
Contig N50	2.43 Mb	68.5 Kb
Contig L50	95	Not available
Anchored to chromosome	926.05 Mb	859 Mb
Mapping back rate of Illumina reads	99.38%	99.83%
Mapping back rate of PacBio reads	98.42%	97.02%

### Repeat annotation

We masked and annotated repetitive sequences and transposable elements (TEs) in the pf3 v1.1 assembly through incorporating *de novo* and homology-based predictions. We built a de novo repeat sequences library by using RepeatModeler v2.0.1^14^, and performed homology-based predictions by using RepeatMasker v4.1.2-p1^15^ with the *Arabidopsis* repeat sequences database. The output cat.gz file (contains list and alignment of repeat regions found in the assembly) of *de novo* and homology-based prediction was merged together and subjected to post-process with RepeatMasker package to produce the final repeat annotation. In total, we identified 770.68 Mb repetitive sequences in the pf3 v1.1 assembly, accounting for 78.99% of its total length (Fig. 3b and Table 3).

**Table 3:** Repetitive element annotation statistics.

Type			Number of elements	Sequence length (bp)	Percentage of genome (%)
Retroelements	LTR elements	Ty1/Copia	321,746	349,077,929	35.78
		Gypsy/DIRS1	151,055	161,352,869	16.54
		BEL/Pao	1,784	570,120	0.06
		Retroviral	26	3,718	0.00
	Non-LTR elements	SINEs	2,843	391,133	0.04
		LINEs	20,245	9,543,583	0.98
	Total of retroelements		523,013	530,076,328	54.33
DNA transposons			122,330	46,900,551	4.81
Rolling-circles			1,503	357,023	0.04
Unclassified			691,910	167,567,353	17.18
Small RNA			7,770	3,151,987	0.32
Satellites			1,611	418,545	0.04
Simple repeats			422,510	17,587,033	1.80
Low complexity			102,295	4,996,624	0.51
Total			1,872,942	770,677,817	78.99

Note: The “Total of retroelements” and the “Total” number is calculated from the total number of bases masked as repeats in the query sequence, and it is not exactly equal to the sum of sub-classes and the sum all classes.

### Gene prediction and functional annotation of the genome

We conducted protein-coding gene prediction with integration of evidence data from *ab initio* training, transcript and homologous proteins alignment through BRAKER pipeline v2.1.6^16^. We prepared transcript evidence by mapping the full-length RNA-seq and short-read RNA-seq data to pf3 v1.1 assembly by using minimap2 v2.23-r1111^17^ and STAR v2.7.9a^18^ respectively, followed by sorting alignment bam files with samtools v1.7^19^. In running the pipeline, the soft-masked pf3 v1.1 assembly was used as input genome (–genome option), the sorted RNA-seq alignment files were used as RNA-seq evidence (–bam option), and peptides of the wax gourd B227 assembly and other three cucurbit species (*Cucumis sativus* ChineseLong v3, *Lagenaria siceraria* v1 and *Cucurbita moschata* v1) were used as homologous protein data (–prot_seq option) and aligned to the genome using GenomeThreader v1.7.0^20^ (–prg gth). Briefly, the pipeline started with generating seed genes by GeneMark-ES v4.69_lic with supported by RNA-Seq evidence. Subsequently it performed *ab initio* training of AUGUSTUS v3.4.0^21^ with the seed genes, RNA-Seq and protein alignment information. Finally, it conducted gene prediction with AUGUSTUS through integrating of the training output, RNA-Seq and homologous protein alignment information. After filtered out genes encoding protein sequence shorter than 50 aa (amino acids), and genes containing internal stop codon, illegal start or stop codon, a total of 37,092 genes were annotated in the pf3 v1.1 assembly (Fig. 3c and Table 4).

**Table 4:** Summary of gene annotation.

Type	pf3 v1.1
Number of genes	37,092
Total gene length	114.70 Mb
Longest gene	118,234 bp
Average gene length	3,089 bp
Number (and percentage) of genes on chromosomes	33,384 (90.00%)
Number of mRNAs	41,101
Total mRNA length	141.33 Mb
Mean exons per mRNA	4.6
Total exon length	40.50 Mb
Number of gene annotated by InterProScan	31,562
Number of gene assigned to a specific GO term	22,709

Furthermore, we performed functional annotation of the predicted genes through searching proteins against InterPro database (v88.0). We carried out this by submitted protein sequences to InterProScan 5 webservice^22^ via a perl script (iprscan5_lwp-nodie.pl) with default parameters. We further used PANNZER2^23^ and eggNOG-mapper v2.1.7^24^ to annotate proteins by Gene Ontology (GO) terms. In total, 31,562 genes were functionally annotated, and 22,707 genes were assigned to specific GO term (Table 4).

### Comparison of the assembly of B227 and pf3

**Fig. 4:**
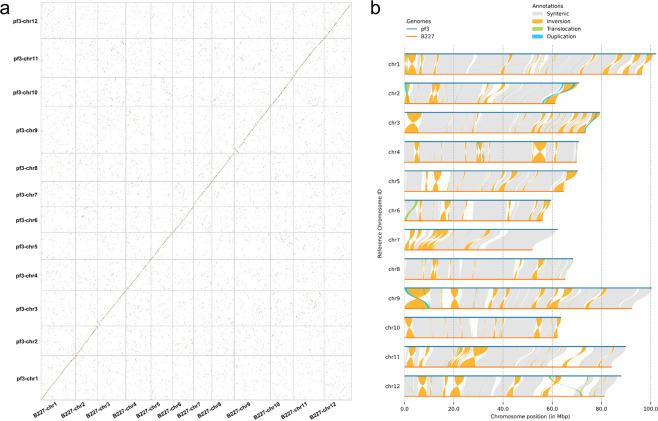
Whole-genome comparison of the pf3 v1.1 with B227 assembly. (**a**) Dot plot for the syntenic blocks. (**b**) Chromosome-level local sequence differences.

To infer the synteny and colinearity the pf3 v1.1 assembly and B227 assembly, we ran the Quick Genome Dot Plot plugin (parameters: Blast e-value 1e-3, Num of BlastHits 5) embedded in TBtools v1.098726^25^. We found that the two assemblies were quite syntenic (Fig. 4a), but there were large-scale inversions at the ends of some chromosomes. We further analysed the genomic rearrangements and local sequence differences between the two assemblies by using the SyRI v1.6^26^ software. We identified a lot of Mb-sized structural rearrangements including inversions, translocations and duplications, and these rearrangements were mainly located at the end of chromosomes (Fig. 4b).

Considering the close genetic background between the two cultivars, and that the pseudo-chromosome of B227 assembly was developed based on a genetic map, it could be deduced that most of the rearrangements may be not real existing but rather mis-oriented or mis-placed in the B227 assembly. To check this, we further split the B227 assembly and re-constructed scaffolds (pseudo-chromosomes) by using Hi-C data of a genetically very close cultivar B418 that also belongs to C2 group. We detected much higher collinearity between the Hi-C-based B227 assembly and the pf3 v1.1, and most of large rearrangements discovered previously disappeared (Supplementary Fig. S1). We also examined the differences between the Hi-C based and genetic map based assemblies of the B227, and detected several large inversions at end of chromosomes (Supplementary Fig. S2). These evidences suggest that most of rearrangements showed in the Fig. 4d are indeed errors in the genetic map based B227 assembly.

### Discovery of genomic variations

To explore genetic variation pattern of the wax gourd germplasm referenced to the newly developed pf3 genome, 31 representative wax gourd accessions (Supplementary Table S1) that we sequenced previously were selected and subjected to mapping and variation discovery procedure. Sequencing data of each accession was mapped onto the pf3 v1.1 assembly by using bwa v0.7.17-r1188^27^ with default parameters, and the alignment files were sorted and indexed with samtools. We called variations of all the 31 accessions together by using bcftools v1.8^28^ with *mpileup* and *call* commands. We applied pre-call filtering with -q 30 and -Q 20 to skip poor mapped reads and low-quality bases when run *mpileup* command, resulting in an initial total of 36,401,973 variant sites. We evaluated summary metrics of the raw variants, and then filtered them by using VCFtools v0.1.16^29^ and bcftools based on quality score, depth, average mapping quality and other criteria as described in the Supplementary Note 2. We finally got more than 12 million high-quality variations, including 12,366,466 single-nucleotide polymorphisms (SNPs) and 286,201 small insertions and deletions (InDels). The distribution of SNPs across the pseudo-chromosomes was as shown in the Fig. 3d. Furthermore, we investigated numbers of SNPs in subset of samples, we found that the C2 group contained the minimum SNPs among the four groups (Table 5), and there was only 593,107 SNPs between B227 and pf3.

**Table 5:** Number of SNPs in subset of samples.

Sample	Number of samples	Number of SNPs
B227	1	593,107
B418	1	653,697
C2 group (including B227 and B418)	11	930,918
C1 group	7	1,288,797
L group	7	1,727,919
W group	6	11,646,942
All	31	12,366,466

## Data Records

The sequencing data, genome assembly and annotation data reported in this paper have been deposited in the Genome Warehouse in National Genomics Data Center (NGDC), Beijing Institute of Genomics, Chinese Academy of Sciences/China National Center for Bioinformation^30,31^, under the BioProject accession number PRJCA010475 that is publicly accessible at https://ngdc.cncb.ac.cn/gwh. All the clean genome sequencing data including PacBio long-read, Illumina short-read and Hi-C data, as well as RNA sequencing data including Nanopore full-length RNA-seq and Illumina short-read RNA-seq data, were deposited in the Genome Sequence Archive (GSA) of NGDC under the accession number CRA007486. The pf3 v1.1 assembly and annotation data have been deposited in the Genome Assembly Sequences and Annotations (GWH) of NGDC under accession number GWHBJVO00000000. The DNA and RNA sequencing data were also submitted to the National Center for Biotechnology Information (NCBI) SRA database with accession number SRR23081782, SRR23081783, SRR23081784, SRR23081781 and SRR23096591 under BioProject PRJNA898819^32–36^. The genome assembly has also been deposited at DDBJ/ENA/GenBank under the accession GCA_027475165.1^37^. Sequencing data of the 30 wax gourd cultivars used for genetic variation discovery are available in the GSA under project accession number PRJCA001140, and sequencing information for these cultivars is summarized in Supplementary Table S1.

## Technical Validation

### Assessment of the genome assembly

To evaluate the completeness of the wax gourd pf3 v1.1 assembly, we first mapped Illumina short-read and PacBio long-reads data back to the assembly, and analysed the alignment file with Qualimap v.2.2.2^38^. The mapping rate of both libraries was above 98% (Table 2), and more than 96.5% of the assembly have at least 20× coverage of Illumina short-read and PacBio long-reads respectively. We then performed the Benchmarking Universal Single-Copy Orthologs (BUSCO) v5.2.2^39^ with eudicots dataset (n = 2,326) to assess the completeness of the assembly. We identified 2,280 complete BUSCOs (98.02%) out of the 2,326 BUSCO groups, including 2,183 complete and single-copy BUSCOs and 97 complete and duplicated BUSCOs (Table 6). The number of fragmented BUSCOs and missing BUSCOs was 9 (0.4%) and 37 (1.5%), respectively.

**Table 6:** BUSCO assessment results.

BUSCO type	Count	Ratio (%)
Complete BUSCOs	2,280	98.02
Complete and single-copy BUSCOs (S)	2,183	93.85
Complete and duplicated BUSCOs (D)	97	4.17
Fragmented BUSCOs (F)	9	0.39
Missing BUSCOs (M)	37	1.59
Total BUSCO groups searched	2,326	100.00

**Fig. 5:**
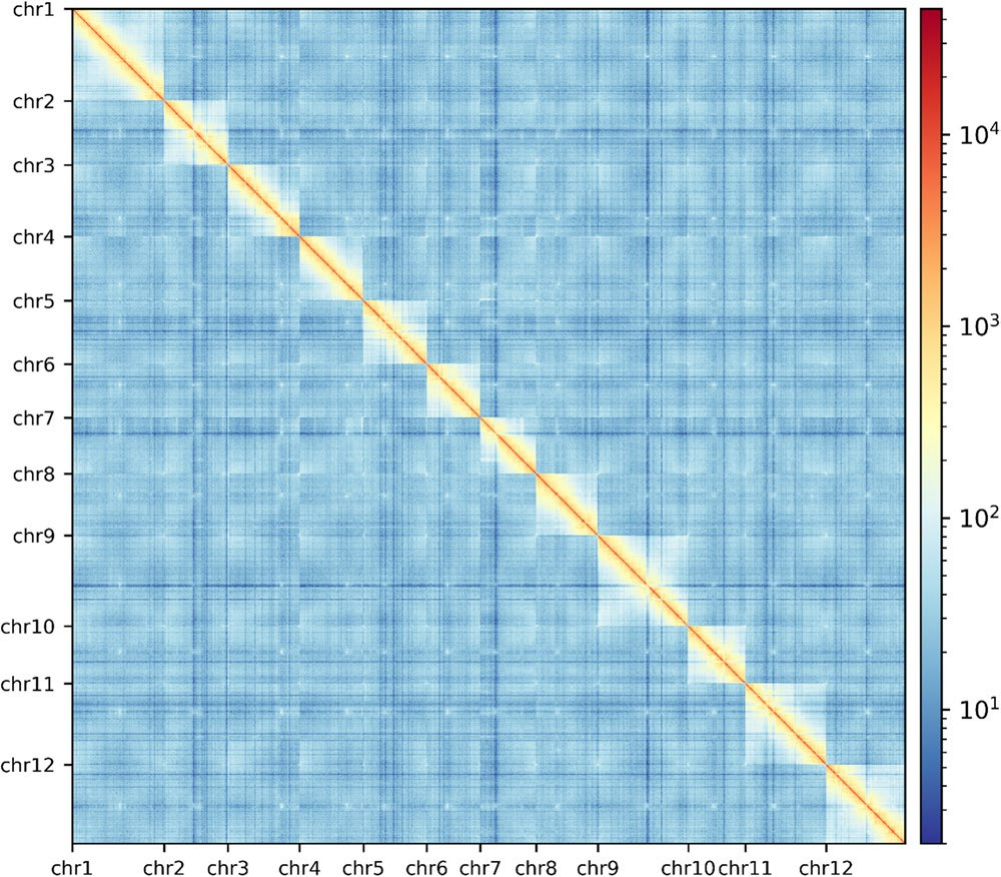
Hi-C contact map of the chromosome-level assembly of pf3. The intensity of interactions was calculated using a bin size of 10 K.

Moreover, we evaluated the result of Hi-C based pseudo-chromosomes construction. We mapped the Hi-C data to the 12 pseudo-chromosomes, and then analysed and visualized with Hicexplorer v3.7^40^. As the heat-map of Hi-C contact displays in Fig. 5, the signal intensities of interaction between the two bins were clearly divided into 12 distinct groups, indicating the high-quality of the pseudo-chromosomes assembly.

## Supplementary information

Supplementary Information for A chromosome-level reference genome of the wax gourd (Benincasa hispida)

## Data Availability

The versions, settings and options of software tools used in this work are described below, and more detailed explanation is described in the Supplementary Notes section. (1) MaSuRCA: v4.0.9, default parameters; (2) Juicer: v1.6, default parameters; (3) 3D-DNA: v180922, default parameters; (4) Juicebox tool: v1.22.01, default parameters; (5) RepeatModeler: v2.0.1, default parameters+; (6) RepeatMasker: v4.1.2-p1, parameters: -xsmall -gff; (7) BRAKER: v2.1.6, parameters:–species = Benincasa_hispida–softmasking–prg gth–gth2traingenes–AUGUSTUS_ab_initio–gff3; (8) minimap2: v2.23-r1111, parameters: whole-genome alignment: -ax asm5 –eqx; mapping PacBio SMRT reads: -ax map-pb; (9) STAR: v2.7.9a, default parameters; (10) samtools: v1.7, parameters: view command: -bS, sort command: -O BAM; (11) GenomeThreader: v1.7.0, default parameters; (12) GeneMark-ES: v4.69_lic, default parameters; (13) AUGUSTUS: v3.4.0, default parameters; (14) InterProScan: v5.56–88.0, parameters: -dp -f tsv; (15) PANNZER2: web server version, default parameters; (16) eggNOG-mapper: v2.1.7, default parameters; (17) TBtools: v1.098726, creating genome dot plot: Quick Genome Dot Plot plugin: evalue 1e-3 Num of BlastHits 5, creating genome circus plot: Advanced Circos (input data prepared via Fasta Stats, One Step MCScanX, Text Merge for MCScanX and Transformat for Micro-Synteny View) with default paremeters; (18) SyRI: v1.6, default parameters; (19) bwa: v0.7.17-r1188, parameters: mapping reads: mem -M; (20) bcftools: v1.8, parameters: mpileup -Ou -q 30 -Q 20 –p; call -m -Ov; (21) VCFtools: v0.1.16, parameters:–remove-filtered-all–remove-filtered-geno-all–max-missing 1.0–min-alleles 2–max-alleles 2; (22) Qualimap: v.2.2.2, parameters: bamqc; (23) BUSCO: v5.2.2, parameters: -m genome -c 40; (24) Hicexplorer: v3.7, parameters: hicBuildMatrix:–binSize 10000, hicPlotMatrix:–dpi 600.
