# Supplementary material for: A chromosome-level reference genome of the wax gourd (Benincasa hispida)
Source: Sci Data. 2023 Feb 7;10:78. doi: 10.1038/s41597-023-01986-7 (PMC9905507; doi:10.1038/s41597-023-01986-7)
Supplement: Supplementary file 1 — Supplementary Information for A chromosome-level reference genome of the wax gourd (Benincasa hispida) [file 41597_2023_1986_MOESM1_ESM.docx]

**Supplementary Information**

**for**

**A chromosome-level reference genome of the wax gourd (*Benincasa hispida*)**

**AUTHORS**: Wenlong Luo, Jinqiang Yan, Shanwei Luo, Wenrui Liu, Dasen Xie, Biao Jiang

**Contents**

**1. Supplementary Table S1 Information of the 31 wax gourd lines used in variants discovery**

**2. Supplementary Fig. S1 Chromosome-level local sequence differences between the Hi-C based B227 assembly and the pf3 v1.1.**

**3. Supplementary Fig. S2 Chromosome-level local sequence differences between the Hi-C based and genetic map based assemblies of the B227**

**4. Supplementary Notes**

**Supplementary Table S1 Information of the 31 wax gourd lines used in variants discovery**

| cultivar | Origin | Total cleaned ases(Gb) | Group | NGDC accession number |
| --- | --- | --- | --- | --- |
| BF19 | Punjab, India | 20.11 | W | SAMC049563 |
| B260-a | Fukushima-ken, Japan | 22.97 | W | SAMC049626 |
| F3 | Self created material | 23.49 | W | SAMC049616 |
| HF3-b | Self created material | 19.37 | W | SAMC049682 |
| HF3-d | Self created material | 17.66 | W | SAMC049684 |
| HB260-b | Self created material | 20 | W | SAMC049680 |
| BN1603 | Puer, Yunnan, China | 24.72 | L | SAMC049665 |
| S15 | Taiwan, China | 19.56 | L | SAMC049699 |
| BN1615 | Lancang, Yunnan, China | 20.8 | L | SAMC049668 |
| BN40 | Jinghong, Yunnan, China | 23.11 | L | SAMC049613 |
| B421 | Quang Tri, Tinh, Vietnam | 20.37 | L | SAMC049594 |
| BN35 | Jinghong, Yunnan, China | 23.53 | L | SAMC049612 |
| BN1620 | Jinghong, Yunnan, China | 19.57 | L | SAMC049669 |
| P73 | Haikou, Hainan, China | 22.96 | C1 | SAMC049689 |
| P126 | Huangmei, Hubei, China | 22.91 | C1 | SAMC049686 |
| B242 | Beijing, China | 20.8 | C1 | SAMC049624 |
| A39FA | Self created material | 23.66 | C1 | SAMC049622 |
| LY1 | ningyang, Shandong, China | 21.48 | C1 | SAMC049685 |
| H10 | Jiangmen, Guangdong, China | 22.46 | C1 | SAMC049677 |
| P86 | Dongguan, Guangdong, China | 21.1 | C1 | SAMC049693 |
| BS529 | Sanshui, Guangdong, China | 24.16 | C2 | SAMC049671 |
| B498 | Jiexi, Guangdong, China | 27.39 | C2 | SAMC049652 |
| B452 | Dongguan, Guangdong, China | 17.39 | C2 | SAMC049640 |
| B506 | Changsha, Hunan, China | 23.91 | C2 | SAMC049658 |
| B514 | Nanning, Guangxi, China | 18.58 | C2 | SAMC049661 |
| B500 | Jiexi, Guangdong, China | 22.62 | C2 | SAMC049654 |
| B418 | Huizhou, Guangdong, China | 23.44 | C2 | SAMC049593 |
| B478 | Guangzhou, Guangdong, China | 22.52 | C2 | SAMC049641 |
| B98-3 | Taishan, Guangdong, China | 20.71 | C2 | SAMC049611 |
| B528 | Sanshui, Guangdong, China | 15.25 | C2 | SAMC049609 |
| B227 | Guangzhou, Guangdong, China | 18.62 | C2 | SAMC049571 |

**
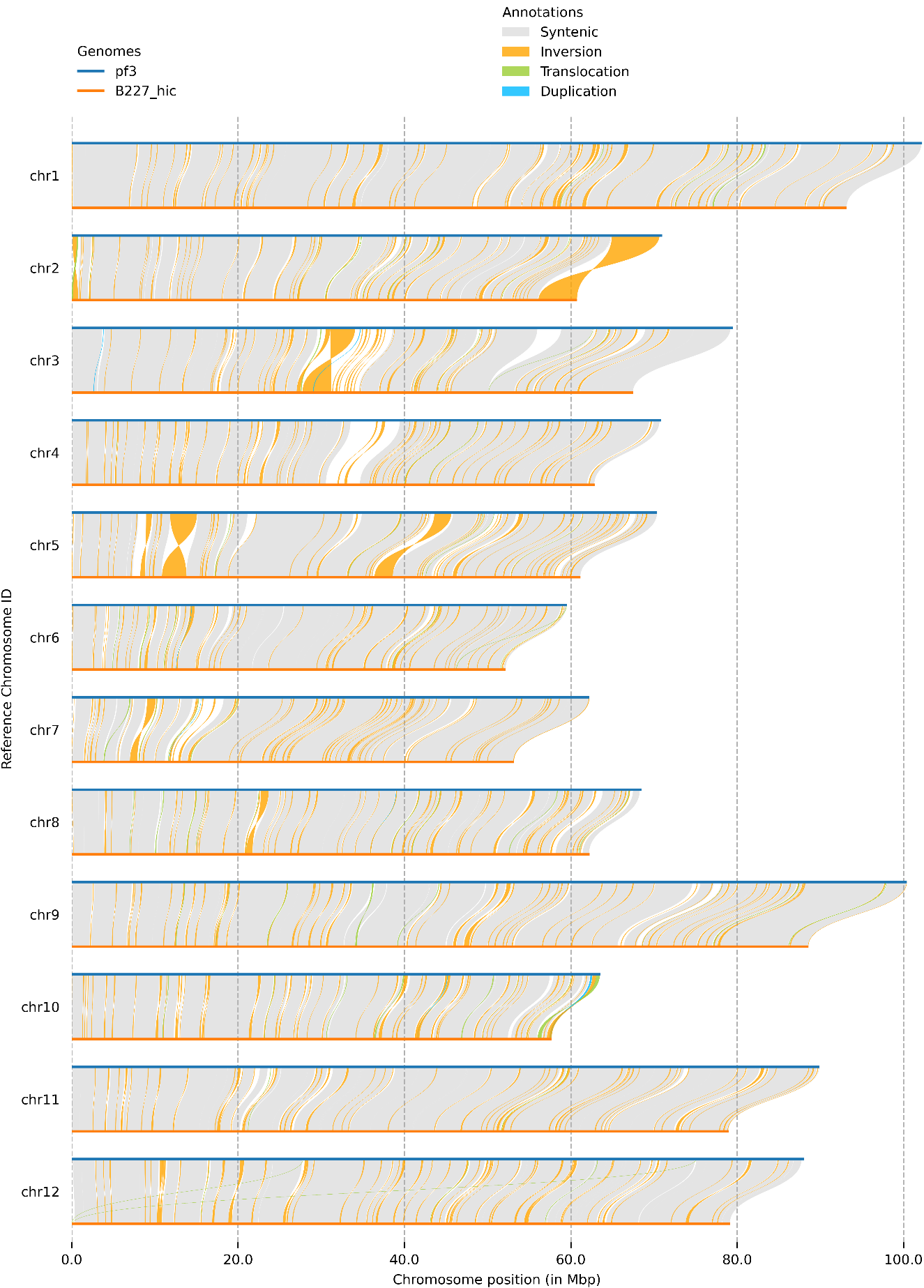
**

**Supplementary Fig. S1 Chromosome-level local sequence differences between the Hi-C based B227 assembly and the pf3 v1.1.**


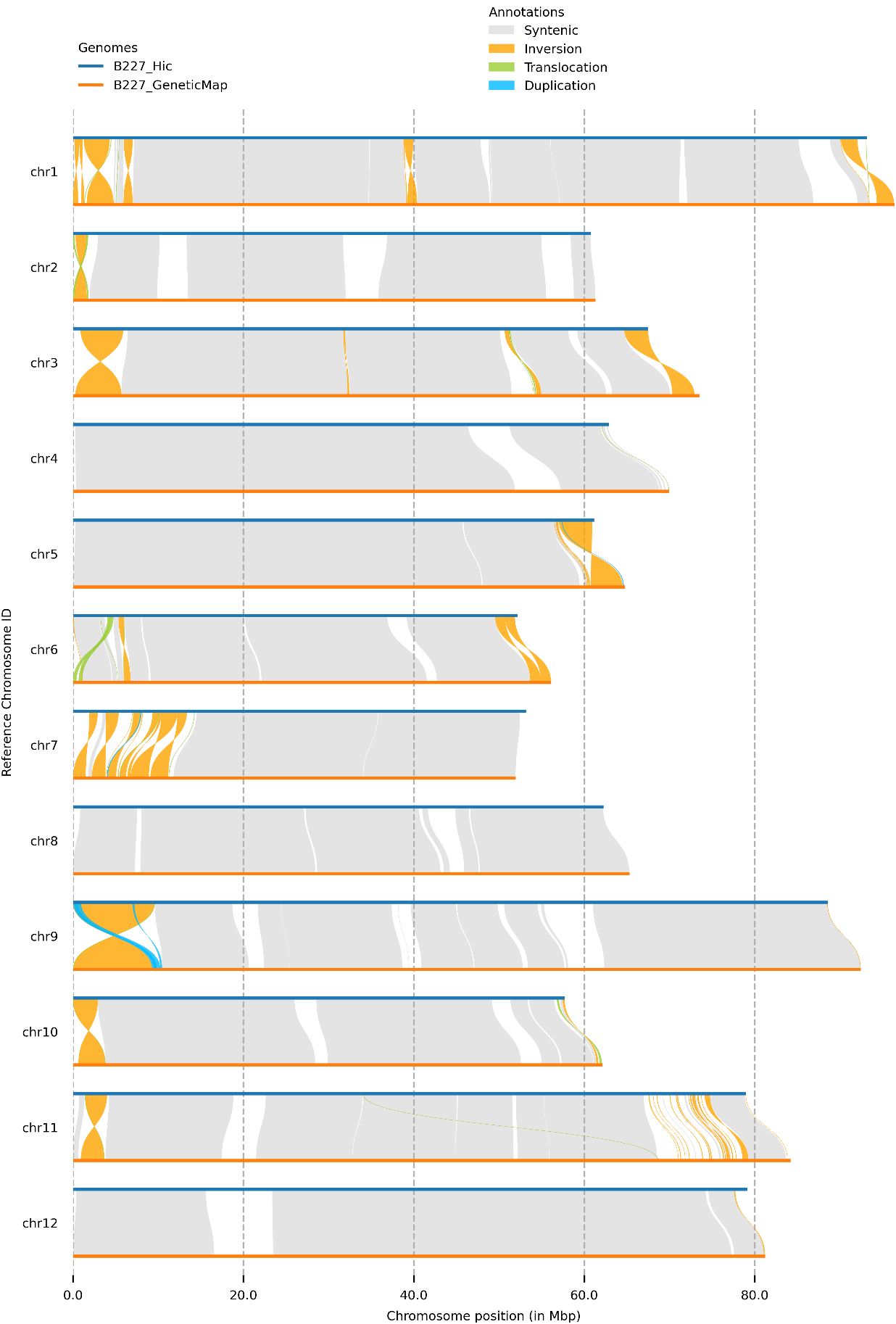


**Supplementary Fig. S2 Chromosome-level local sequence differences between the Hi-C based and genetic map based assemblies of the B227**

**Supplementary Notes**

**Note 1: Genome assembly and repeat annotation**

We have Illumina short reads (approx. 50-fold), PacBio long reads (approx. 86-fold) and Hi-C data, so we decided to use hybrid assembly strategy. We chose the MaSuRCA for which is a very flexible and versatile assembler combines the benefits of deBruijn graph and Overlap-Layout-Consensus assembly algorithms. We ran the MaSuRCA assembler v4.0.911 with default parameters using Illumina data and PacBio data, which generated overlaps, unitig, contig and final scaffold sequences in the output. The unitig sequences, a special kind of contigs that are no competing choices in terms of internal overlaps. The unitigs serve as preliminary, high-confidence, conservative contigs that seed the rest of the assembly pipeline. Due to the use of high-coverage PacBio long reads, we achieved a high continuity of unitigs with N50 size of 2.43 Mb. Therefore, we took the unitigs as initial assembly and generated scaffold by using Hi-C data through 3D-DNA v180922 pipeline. The Hi-C based scaffolds were then visualized and subjected to two round of manual corrections by using the Juicebox tool package v1.22.01, from which the final assembly (pf3 v1.1) was generated.

In the repeat annotation, we firstly performed *de novo* identification of repeats from the pf3 v1.1 assembly using RepeatModeler v2.0.1 with default parameters. We then searched repeats in the assembly through RepeatMasker v4.1.2-p1 by using library of *Arabidopsis* as input. Finally, results from the two steps were analyzed together to produce the final repeat annotation. Classification of repeats was retrieved from Dfam classification using RepeatMasker inner function.

**Note 2: Discovery of genomic variations**

Variant discovery often starts with (I) the quality control of sequencing reads, followed by (II) the mapping of reads to a reference, and then the (III) identification (calling) of variants is performed based on alignments. Finally, filtration of variants to filter out low-quality sites. Many variant discovery pipelines have been developed, and several benchmarking studies been conducted on these pipelines. Though relative low concordance concordance between pipelines, popular variant callers like SAMtools/BCFtools, GATK and FreeBayes generally gives promising performance. The purpose of this study is to discovery high-confidence variants in the genome of re-sequenced wax gourd inbred lines listed the Supplementary Table 1. The task is relatively simple, for we have about 20x depth data for each line, the variants are germline variants and the most majority of them should be homozygous. So we decide to use the BWA-SAMtools/BCFtools pipeline, and filter the initial variants with multi criteria according to its summarized quality metrics.

The detailed steps and the commands are as follows:

1. Mapping reads to ref. (the pf3 v1.1 assembly), sorting and indexing

1) create a reference genome index

*$bwa index ref.fa*

2) align reads of each line to reference with bash script:

*#!/bin/bash*

*for f in $(ls ./*_1.fq.gz)*

*do*

*R1=$f*

*R2=${f/_1.fq.gz/}_2.fq.gz*

*bname=${f/_1.fq.gz/}.aln2pf3*

*rname=${f/_1.fq.gz/}*

*bwa mem -t 64 -R '@RG\tID:$rname\tSM:$rname\tLB:Lib1' ref.fa $R1 $R2 | samtools sort -@ 16 -O BAM -T /tmp -o $bname.bam*

*done*

3) indexing the alignment bam files

*$for f in $(ls ./*_aln2pf3.bam); do samtools index $f; done*

2. Call variants with bcftools and concatenate together

1) create a tab delimited file that contains the target regions with chromosome, start and end position:

*chr1 1 50000000*

*chr1 50000001 102236916*

*chr2 1 50000000*

2) call variants using GNU Parallel, and then concatenate together

*$cat pf3.chr.start.stop.tab | awk 'FS="\t", OFS="" {print $1,":"$2"-"$3}'| parallel –j 40 " bcftools mpileup -b lines_aln2pf3.bam.list -Ou -q 30 -Q 20 -p -f pf3_genome_full_masked.fa -r {} | bcftools call -m -Ou --variants-only -o {}.bcftools.q30Q20p.bcf "*

*$ls *.bcftools.q30Q20p.bcf > bcf.list && bcftools concat -Ov -o lines.bcftools.q30Q20p.vcf -f bcf.list*

**#Key parameters explanation:**

*-q 30*, skip alignments with mapping quality smaller than 30

*-Q 20*, skip bases with base quality smaller than 20

3. Extract high quality SNPs

*$vcftools --vcf lines.bcftools.q30Q20p.vcf --max-missing 1.0 --remove-indels --remove-filtered-all --remove-filtered-geno-all --recode --recode-INFO-all --out lines.bcftools.q30Q20p_NoMissing.SNP*

**#Key parameters explanation:**

*--max-missing 1.0*, no missing data allowed

*--remove-indels*, exclude sites that contain an indel

*--remove-filtered-all*, remove all sites with a FILTER flag other than PASS

*--remove-filtered-geno-all*, excludes all genotypes with a FILTER flag not equal to "." (a missing value) or PASS.

*$* *bcftools filter -g3 -G10 -i 'GT="1/1" && QUAL> 100 && AN=62 && DP>124 && DP<1240 && MQ>40 && MQ0F=0 && RPB>0.001 && MQB>0.001 && BQB>0.001 && MQSB>0.001 ' lines.bcftools.q30Q20p_NoMissing.SNP.recode.vcf \*

*> lines.bcftools.q30Q20p_HighConfHomSNP.vcf*

**#Key parameters explanation:**

*-g 3*, filter SNPs within 3 base pairs of an indel

*-G10*, filter clusters of indels separated by 10 or fewer base pairs allowing only one to pass

*GT="1/1"*, the genotype is homozygous alternate (1/1)

*QUAL> 100*, quality score greater than 100

*AN=62*, allele number equal to 62 (31 samples * 2n)

*DP>124 && DP<1240*, total raw read depth in range between 124 and 1240 (approx. 20-fold depth per sample, range from 31*20*0.2 to 31*20*2)

*MQ>40*, average mapping quality greater than 40

*MQ0F=0*, Fraction of MQ0 reads is zero (smaller is better)

*RPB>0.001*, Mann-Whitney U test of Read Position Bias larger than 0.001 (bigger is better)

*MQB>0.001,* Mann-Whitney U test of Mapping Quality Bias larger than 0.001 (bigger is better)

*BQB>0.001*, Mann-Whitney U test of Base Quality Bias larger than 0.001 (bigger is better)

*MQSB >0.001*, Mann-Whitney U test of Mapping Quality vs Strand Bias larger than 0.001 (bigger is better)

4. Extract high quality indels

*$vcftools --vcf lines.bcftools.q30Q20p.vcf --max-missing 1.0 --keep-only-indels --remove-filtered-all --remove-filtered-geno-all --recode --recode-INFO-all --out lines.bcftools.q30Q20p_NoMissing.indel*

*$bcftools filter -i 'GT="1/1" && QUAL>100 &&IDV>4 && IMF>0.25 && AN=62 && MQ>40 && MQ0F=0 && DP>124 && DP<1240 ' lines.bcftools.q30Q20p.INDEL.vcf > lines.bcftools.q30Q20p_HighConfHomINDEL.vcf*

**#Key parameters explanation:**

*IDV>4, maximum number of reads supporting an indel greater than 4*

*IMF>0.25, maximum fraction of reads supporting an indel greater than 0.25*

5. Extract sub-set of data and get statistics

$*bcftools view -s SAMPLE1 lines.bcftools.q30Q20p_HighConfHomSNP.vcf | bcftools view -i 'GT="1/1" ' > SAMPLE1.bcftools.q30Q20p_HighConfHomSNP.vcf*

$*bcftools view -s SAMPLE1,SAMPLE2 lines.bcftools.q30Q20p_HighConfHomSNP.vcf | bcftools view -i 'GT="1/1" ' > Group1.bcftools.q30Q20p_HighConfHomSNP.vcf*

*$bcftools stats SAMPLE1.bcftools.q30Q20p_HighConfHomSNP.vcf > SAMPLE1.bcftools.q30Q20p_HighConfHomSNP.vcf.stats*

*$bcftools stats -d 0,1240,30 Group1.bcftools.q30Q20p_HighConfHomSNP.vcf > Group1.bcftools.q30Q20p_HighConfHomSNP.vcf.stats*
